# Eating from the same plate? Revisiting the role of labile carbon inputs in the soil food web

**DOI:** 10.1016/j.soilbio.2016.06.023

**Published:** 2016-11

**Authors:** Franciska T. de Vries, Tancredi Caruso

**Affiliations:** a Faculty of Life Sciences, The University of Manchester, Oxford Road, Manchester M13 9PT, United Kingdom; b School of Biological Sciences and Institute for Global Food Security, Queen’s University of Belfast, 97 Lisburn Road, Belfast BT9 7BL, Northern Ireland, United Kingdom

**Keywords:** Bacteria, Food web interactions, Fungi, Modelling, Root exudates, Soil food web

## Abstract

An increasing number of empirical studies are challenging the central fundamentals on which the classical soil food web model is built. This model assumes that bacteria consume labile substrates twice as fast as fungi, and that mycorrhizal fungi do not decompose organic matter. Here, we build on emerging evidence that points to significant consumption of labile C by fungi, and to the ability of ectomycorrhizal fungi to decompose organic matter, to show that labile C constitutes a major and presently underrated source of C for the soil food web. We use a simple model describing the dynamics of a recalcitrant and a labile C pool and their consumption by fungi and bacteria to show that fungal and bacterial populations can coexist in a stable state with large inputs into the labile C pool and a high fungal use of labile C. We propose a new conceptual model for the bottom trophic level of the soil food web, with organic C consisting of a continuous pool rather than two or three distinct pools, and saprotrophic fungi using substantial amounts of labile C. Incorporation of these concepts will increase our understanding of soil food web dynamics and functioning under changing conditions.

## Highlights

• The fundamental assumptions in the classical soil food web are being challenged.
• We argue that labile C forms a large and dynamic C input to the soil food.
• We show that fungi and bacteria can coexist with significant fungal labile C use.
• We propose a new labile C driven conceptual model based on these findings.
• These concepts will increase our understanding of soil food web dynamics.

## Graphical abstract

**Figure:**
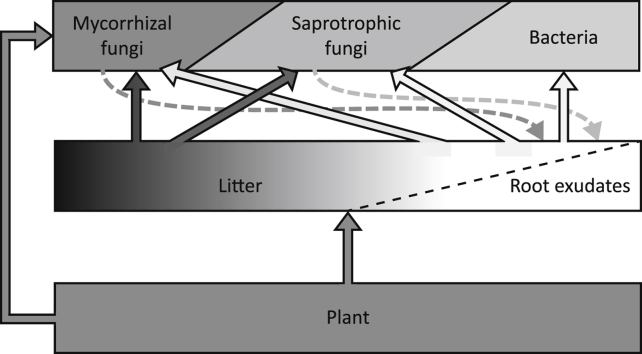

## 1 Introduction

It has long been acknowledged that interactions in the soil food web are crucial for processes of soil carbon (C) and nitrogen (N) cycling. In the first complete soil food web, Hunt et al. (1987), identified the presence of a separate fungal and bacterial energy channel, as well as a root energy channel, formed by saprotrophic fungi and their consumers, bacteria and their consumers, and mycorrhizal fungi and root-feeding nematodes and their consumers, respectively. While the fungal and bacterial energy channels can be considered ‘brown’ because the bottom trophic levels—fungi and bacteria—obtain their energy from dead organic matter (detritus), the root energy channel can be considered ‘green’ because mycorrhizal fungi and root-feeding nematodes obtain their energy directly from living plants. Both modelling and empirical studies have consistently found more efficient C and N cycling in the fungal energy channel than in the bacterial energy channel (Hunt et al., 1987, De Ruiter et al., 1993, De Vries et al., 2011, Holtkamp et al., 2011, De Vries et al., 2012a). In addition, theoretical and empirical work has shown that the presence of a ‘slow’ fungal energy channel with weak interactions strengths, and coupling of the two energy channels by higher-level consumers, confers stability to the soil food web (Rooney et al., 2006, De Vries et al., 2012b, Rooney and McCann, 2012). Shifts in the ratio between the fungal and bacterial energy channel (often measured as the shift in fungal/bacterial biomass ratio) are generally attributed to changes in agricultural management and plant community composition, and consequently in the quantity and quality of organic substrates, which primarily consist of plant inputs, i.e. leaf and root litter and root exudates (Bardgett and McAlister, 1999, Wardle et al., 2004, Bardgett and Wardle, 2010, De Vries et al., 2012d).

Despite its conceptual advances, some of the fundamental assumptions in the classical food web model that support these modelled and observed patterns are now being challenged by an increasing number of experimental and theoretical studies. In particular, evidence is mounting that feeding interactions in the soil are not restricted to the traditional energy channels (e.g. de Boer et al., 2005, Heidemann et al., 2011, Geisen et al., 2015). In addition, the original assumption that mycorrhizal fungi do not decompose organic matter has been revised in recent years by evidence that, in particular, ectomycorrhizal (EM) fungi can decompose organic matter (Read and Perez-Moreno, 2003, Phillips et al., 2013). However, the classical soil food web does not distinguish between arbuscular mycorrhizal (AM) and EM fungi. Moreover, the classical soil food web distinguishes between a labile and a recalcitrant pool of organic matter, and assumes that “bacteria use labile substrates twice as fast, per unit biomass, as do fungi and that (saprotrophic) fungi use resistant substrates twice as fast per unit biomass as do bacteria” (Hunt et al., 1987). This concept of different pools of organic matter has been challenged recently by the argument that soil organic matter forms a continuum of states and pools (Lehmann and Kleber, 2015). Here, we will focus on emerging evidence that challenges the traditional model of C inputs and their use by the bottom trophic levels, and the implications these may have for the traditional soil food web model.

## 2 C flow in soil food webs

The first food web models assumed that the C that fuels the detrital fungal and bacterial energy channels, consisting of a labile and a recalcitrant pool, predominantly originated from aboveground inputs such as leaf litter. This view was updated by Pollierer et al. (2007), who showed that soil fauna predominantly derived their C from root litter and exudates and not from leaf litter. At the time, the prevailing hypothesis was that only bacteria used labile C, and that it was unlikely that the highly labile C in root exudates would contribute energy to the fungal energy channel and higher tropic levels. However, recent work shows that root exudates constitute a major pathway of belowground C inputs (Nguyen, 2009) and are fundamental to food web controls on C and N cycling in response to climate change (Phillips et al., 2011). Moreover, recent evidence shows that both bacteria and fungi rapidly consume and respire root exudate C (De Deyn et al., 2011, Rousk and Frey, 2015), thus challenging the view that fungi primarily consume recalcitrant litter. Supporting these findings, Eissfeller et al. (2013) found root-derived recent photosynthate C in higher trophic levels of both the fungal and the bacterial energy channel.

Another important source of belowground labile C inputs is the transfer of recent plant photosynthate C to mycorrhizal fungal hyphae, which can occur extremely quickly (De Deyn et al., 2011, Hannula et al., 2012). Although it is assumed that the ability of AM fungi to decompose organic matter is limited, EM fungi have been shown to be able to decompose or cleave organic substrates to meet their nutrient demand (Read and Perez-Moreno, 2003, Talbot et al., 2008, Cheng et al., 2012). Recent work shows that this ability of EM fungi to decompose organic matter can increase soil C pools through competition for organic N between EM fungi and the decomposer community, supposedly resulting in a reduction in soil organic matter nutrient concentrations and increased soil C inputs through greater plant growth (Orwin et al., 2011, Averill et al., 2014). While empirical mechanistic research into the exact mechanisms underlying this increase in soil C pools is sorely needed, EM decomposition of organic matter also has the potential to increase the availability of labile substrates for bacteria and fungi and the energy channels they support (sensu Moore et al., 2004). In addition, AM fungi can prime the decomposition of organic matter by supplying plant-derived C to saprotrophic fungi and bacteria (Herman et al., 2012). Thus, the root energy channel can contribute to the labile C pool that is used by fungi and bacteria via two mechanisms: decomposition of organic matter by EM fungi, and direct transfer of recent root-derived photosynthate C by AM fungi. Importantly, this root-derived C in AM hyphae can enter this labile litter pool relatively quickly, for example when hyphae are pierced by fungal-feeding nematodes, similar to bacteria and fungi leaking their internal solutes as a waste product of grazing (Hunt et al., 1987, Koller et al., 2013). Therefore, AM fungi can connect the three energy channels at the bottom of the soil food web, providing a rapid pathway through which recently photosynthesised C enters the soil food web.

## 3 A new central role for labile C and its consumption in soil food webs

**Fig. 1:**
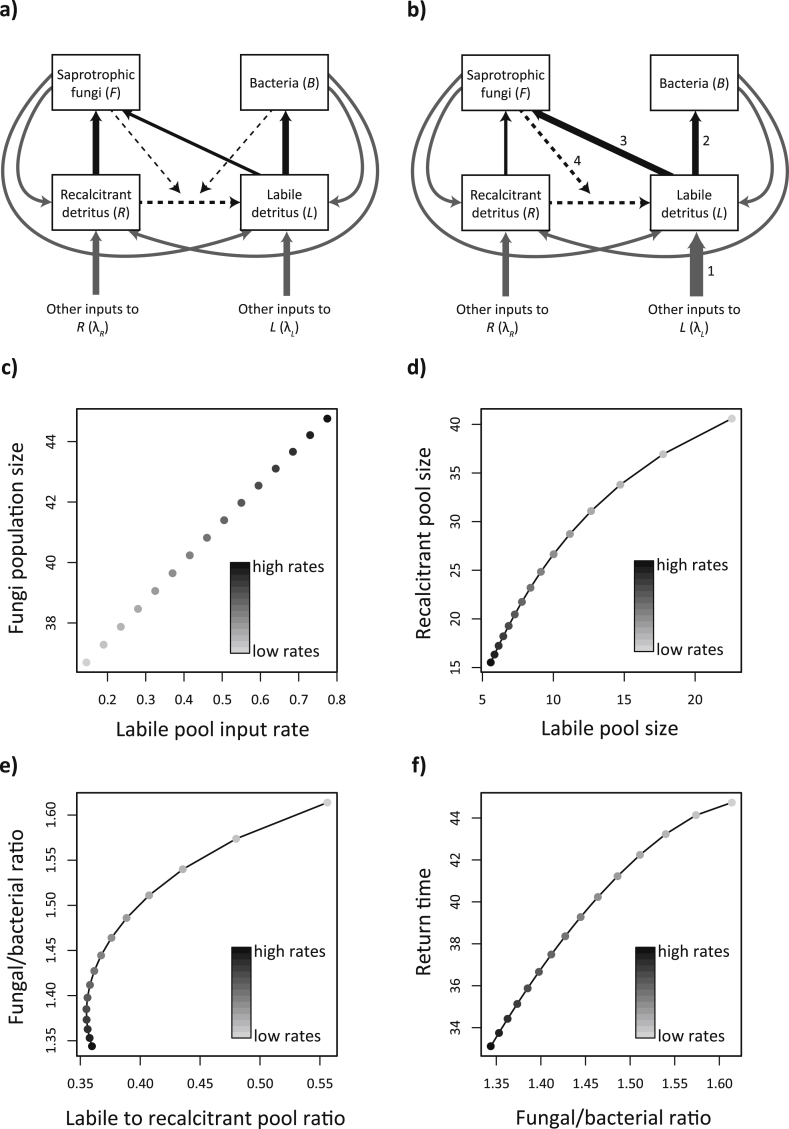
Simple model incorporating the effects of two C pools on saprotrophic fungal and bacterial dynamics, as in Moore et al. (2004). Grey arrows represent the creation of new detritus from external and internal sources, black arrows represent the flow of detritus derived energy, and the dashed line represents ontogenetic change of detritus and the effects fungi and bacteria have on this process. In the original model (a), fungi mostly consumed recalcitrant material, supplemented by a small amount of labile detritus, while bacteria only consumed labile detritus. In our proposed model (b), inputs into the labile detritus pool are increased, as well as fungal consumption of this pool (note that in our model, the arrow via which bacteria affect the ontogenetic production of labile C from recalcitrant C has been removed, since no bacterial consumption of recalcitrant litter exists in the model). After initial model exploration, (see Supplementary Methods), we created a number of scenarios to investigate the behaviour of the model under our proposed modifications. In panels c to f, each dot shows the long term equilibrium value of one scenario. Scenarios differ only for four parameters (which all consist of intrinsic rates per unit biomass): the labile pool input rate (arrow 1), the labile pool consumption rate of both bacteria (arrow 2) and fungi (arrow 3), and the transfer rate of material from the recalcitrant to the labile pool by fungi (arrow 4). Low rates scenarios (light shades) had low values for all four parameters, while high rate scenarios (dark shades) had high values: as the external input to the labile pool increases, both fungi and bacteria increase the consumption rate of the labile pool. Fungi can achieve high consumption of the labile pool while also consuming the recalcitrant pool. These increased rates also correspond to increased fungal mediated transfers from the recalcitrant to the labile pools. The scenarios were designed to cover a broad range of parameter variation while keeping the ratios within reasonable values. Fungi increase with increased input to the labile pool (c) but bacteria do too as shown by the variation of the fungal to bacterial ratio in relation to the pools ratio (d and e). These dynamics also create a positive relationship between the fungal to bacterial ratio and return time to equilibrium after perturbations (f), implying that communities dominated by fungi are less resilient to perturbation than those dominated by bacteria. For all parameters and modelling details see Supplementary Methods.

Despite its relatively small pool size, fluxes of labile (dissolved organic) C are large because of continuous production (through decomposition and root exudation) and consumption (van Hees et al., 2005, Boddy et al., 2007). For example, van Hees et al. (2005) estimated that heterotrophic respiration of root exudate C constitutes 10–20% of total soil respiration. Despite slight modifications in soil food web models to represent the complex role of C inputs (e.g. the inclusion of a water soluble sugar pool in Holtkamp et al. (2008)), current food web models do not represent the importance of this C pool, and its consumption by the bottom trophic levels of the soil food web. Here, we propose the following modifications to existing food web models:

1. Despite the usually small standing pool size of labile C, inputs of labile C are the dominant source of C for the bottom trophic levels in the soil food web on short to medium timescales (hours to seasons) (Bardgett et al., 2005).
2. Saprotrophic fungi use more labile C than previously assumed. Using the model from Moore et al. (2004) (Fig. 1a), we show that fungal and bacterial populations can coexist in a stable state with large inputs into the labile C pool, a high fungal use of labile C, and high fungal mediated transfer of C from the recalcitrant to the labile pool (Fig. 1, Supplementary Methods). By increasing inputs into the labile C pool and the consumption of this pool by fungi (Fig. 1b), we show that fungi can achieve high consumption of the labile pool while also consuming the recalcitrant pool. Our model shows that both fungi and bacteria increase with increased input to the labile pool and increased rates of fungal mediated transfer of labile C (Fig. 1c, e).
3. In addition to their well-established role in protecting soil C through increasing soil aggregation (Rillig and Mummey, 2006, Wilson et al., 2009), EM fungi can decompose organic matter to meet their nutrient demands, thereby potentially increasing the amount of labile substrate available for saprotrophic fungi and bacteria.
4. The role of labile C is enhanced further through the contribution of mycorrhizal fungi to this C pool, through EM fungal organic matter decomposition by extracellular enzymes, and through AM fungi supplying saprotrophic fungi and bacteria with recent photosynthate.

This proposed role of labile C as a major C input into the soil food web has important implications for our understanding of soil C cycling and the role of the soil food web. Intuitively, the high use of labile C by saprotrophic fungi and bacteria might result in higher microbial turnover and respiration, priming of the decomposition of soil organic matter, and lower soil C pools. However, following the Microbial Efficiency-Matrix Stabilization (MEMS) framework by Cotrufo et al. (2013), labile C substrates are used more efficiently by microbes than recalcitrant litter, and are thus the most important source of microbial products and the main precursor of stable soil organic matter. Our proposed modifications are in line with the MEMS framework and suggest a higher microbial C use efficiency of labile C substrates than previously assumed. In particular, EM decomposition of organic matter would increase the availability of labile C, and high labile C use by fungi would increase soil organic matter formation because of their intrinsically higher substrate use efficiency compared to bacteria (Six et al., 2006; but see Thiet et al., 2006). These ideas also support the idea that soil organic matter consists of a continuum of states that are continuously processed by decomposers, and highlight the need to put a greater emphasis on C fluxes than stocks (Lehmann and Kleber, 2015).

This prominent role of labile C use by the bottom trophic levels has implications for higher trophic levels in the soil food web and soil food web dynamics. Root exudates are an important constituent of the labile C pool in soil, and form a continuous but highly dynamic C source, in contrast to the pulsed, seasonal, but slowly fluctuating supply of leaf and root litter. The release of root exudates and the transport of plant C to mycorrhizal hyphae are tightly coupled with plant photosynthetic activity (Heinemeyer et al., 2006), and fluctuate with changes in temperature, moisture, and light availability. Thus, populations and communities of fungi and bacteria that use labile C as their main C source will fluctuate over short, within-seasonal, timescales (Bardgett et al., 2005) and, as a consequence, so might higher trophic levels that feed on them (Moore et al., 2014). In addition, the diffusion of exudates from roots into the soil is highly spatially patterned and results in hotspots of microbial populations and their consumers. Importantly, higher fungal consumption of labile C might result in a more homogenous distribution of C in soil through translocation of this C via hyphal networks, as shown by Muller et al. (2016).

High use of labile C in the form of root exudates by bacteria and fungi also has the potential to affect trophic interactions in the soil food web. Where the classical models assumed that fungi and bacteria mostly consume particulate organic matter, the inclusion of an additional significant labile C pool would increase microbial populations (see Fig. 1). While the importance of fungi for supplying bacteria with labile C has been recognised (Moore et al., 2004, de Boer et al., 2005), higher fungal consumption of labile C would increase competition between fungi and bacteria. In our model (Fig. 1) we show that bacterial and fungal populations can coexist under these scenarios. This coexistence might be facilitated by antifungal strategies employed by bacteria (de Boer et al., 2005), or through spatial or temporal niche separation. For example, it is well known that fungi are more resistant to drought than bacteria (De Vries et al., 2012b, Guhr et al., 2015), and might thus outcompete bacteria for labile C during dry spells, or in dry microsites. In addition, the spatial patterning of high inputs of labile C, as is the case with root exudates, has been shown to promote top-down control of microbial populations by their consumers (Moore et al., 2014). Importantly, microbial grazers can affect levels of labile C by affecting biomass and activity of microbial prey (Moore et al., 2014). These mechanisms likely form a negative feedback to keep increasing fungal and bacterial populations, as found in our model without microbial grazers, in check.

The increased use of root exudates by fungi and bacteria has important implications for the response of soil food webs to disturbance. Soil food web recovery after a disturbance typically occurs from the bottom up and has been shown to be positively affected by the quantity of labile belowground plant C inputs (De Vries et al., 2012c, De Vries and Shade, 2013). It is well known that root exudation increases under elevated atmospheric concentrations of CO_2_, but plant physiological processes also rapidly respond to disturbances such as changes in temperature and moisture, thereby affecting belowground response (Bardgett et al., 2013). For example, it has been shown that warming stimulates root exudation, which in turn stimulated microbial activity (Yin et al., 2013). Therefore, plant physiological response to disturbance likely has an equally important effect on belowground response as shifts in plant community composition (Bardgett et al., 2013). Importantly, our modelled results support the general notion that fungi recover slower after a perturbation than do bacteria (Fig. 1f). Thus, if the labile C pool drives soil food web dynamics, aboveground-belowground linkages are stronger than previously assumed, especially on short timescales, and soil food web dynamics will have stronger and more immediate feedbacks to global change dynamics.

## 4 Conclusion

**Fig. 2:**
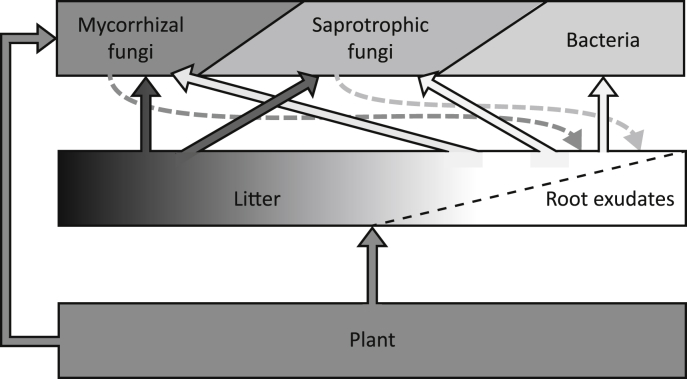
Conceptual model depicting the role of C inputs for the bottom trophic level of the soil food web, and the overlapping, fluent C use abilities of mycorrhizal fungi, saprotrophic fungi, and bacteria. Shading of boxes and arrows indicates the quality of C, with lighter shades indicating highly labile C. Solid arrows indicate C flow. Dashed arrows indicate mycorrhizal and decomposer fungi mediated decomposition and transfer of labile C (see text for details on the different mechanisms). Note that for clarity, no distinction is made between ectomycorrhizal fungi and arbuscular mycorrhizal fungi.

We propose a new conceptual model, in which labile C inputs, and specifically root exudates, form a significant C source for the bottom trophic level of the soil food web. Here, organic C inputs consist of a continuous pool rather than two or three distinct pools, and saprotrophic fungi use substantial amounts of labile C (Fig. 2). Modelling organic C input and quality as a continuous pool may have far reaching consequences for C dynamics, for example in terms of temperature dependence of decay rate under climate change scenarios (Bosatta and Agren, 1999, Agren and Bosatta, 2002, Lehmann and Kleber, 2015). EM fungi in particular are able to decompose organic matter and contribute to the labile C pool, while AM fungi can transport recent plant-derived labile C back into it and prime the decomposition of recalcitrant organic matter. These findings give scope to a trait-based rather than a taxonomic separation of functional groups in the soil food web. While much work is underway classifying microbes and their consumers on the basis of their functional traits (e.g. Crowther et al., 2014, Treseder and Lennon, 2015, da Silva et al., 2016), and feeding relationships in the soil food web are being revised (see other articles in this issue), a detailed understanding of these traits is needed to revise the classical soil food web. To validate our proposed role of root exudates for the soil food web, measurements of root exudation and soil food web C use and population dynamics are needed at high temporal resolution. Although not an easy task, novel methods such as metabolomics, compound-specific isotope ratio analyses, and high-throughput sequencing and barcoding approaches are available to facilitate this. Incorporation of the concepts we propose here in existing food web models will increase our understanding of mechanistic links between aboveground communities and soil food webs, with implications for soil food web dynamics and functioning under changing conditions.
