# Supplementary material for: Eating from the same plate? Revisiting the role of labile carbon inputs in the soil food web
Source: Soil Biol Biochem. 2016 Nov;102:4–9. doi: 10.1016/j.soilbio.2016.06.023 (PMC5061327; doi:10.1016/j.soilbio.2016.06.023)
Supplement: Supplementary file 1 [file mmc1.docx]

**Supplementary Methods**

The model (Fig. 1a,b) is based on Moore et al. (2004) and describes detrital dynamics in terms of a recalcitrant and a labile pool (*R* and *L*), the consumption of these two pools by fungi (*F*), and the consumption of the labile pool by bacteria (*B*). The model also accounts for the fact that fungi and bacteria directly contribute to the detrital pools, with fungi also transferring materials from the recalcitrant to the labile pool. Given our purpose and with a minor loss of generality, we simplified the original formulation by Moore et al. ([2004](#_ENREF_5)) by: i) removing the effects of weathering and leaching; and ii) creating some compound parameters that implicitly account for all the aspects related to detritus consumption (consumption rate, assimilation and yield efficiency). However, the fundamental pathways depicted for this model in Figure 6 of Moore et al. (2004) are also represented in our model.

The system of differential equations of our model is:

$$\frac{dR}{dt}=\lambda_{R}+{a d}_{B} B+{b d}_{F} F-\alpha F R-cr_{F} F R$$

$$\frac{dL}{dt}=\lambda_{L}+{\left( 1-a \right) d}_{B} B+{\left( 1-b \right) d}_{F} F+\alpha F R-dr_{FL} F L-er_{B}B L$$

$$\frac{dF}{dt}=r_{F}R F+r_{FL} L F-d_{F} F-m_{F} F^{2}$$

$$\frac{dB}{dt}=r_{B} L B-d_{B} B-m_{B} B^{2}$$

where i) $\lambda_{L}$and $\lambda_{R}$are rates of external input to R and L, ii) $r_{F}$, $r_{B}$, $r_{FL}$ , are compound parameters accounting for consumption rate, assimilation and yield efficiency, iii) a, b, c, d and e are compound parameters accounting for the positive and negative contribution of *F* and *B* to *R* and *L*, iv) $d_{F}$ and $d_{B}$ are the mortality rates, v) $m_{F}$ and $m_{B}$ are implicit, phenomenological logistic terms, and vi) $\alpha$ is the rate of transfer from *R* to *L* by fungi.

The Jacobian ([Hartman, 1973](#_ENREF_3); [Hale, 1980](#_ENREF_2)) J of the system is:

|  | R | L | F | B |
| --- | --- | --- | --- | --- |
| R | $-\alpha F- c r_{F} F$ | 0 | $b d_{F}-\alpha R - c r_{F}R$ | ${a d}_{B}$ |
| L | $\alpha F$ | $-d r_{FL}F- e r_{B} B$ | $\left( 1 - b \right)d_{F}+\alpha R - d r_{FL}L$ | $(1 - a) d_{B} - e r_{B} L$ |
| F | $r_{F} F$ | $r_{FL} F$ | $r_{F} R+ r_{FL} L-d_{F}-{2m}_{F} F$ | 0 |
| B | 0 | $r_{B} B$ | 0 | $r_{B} L-d_{B}-{2m}_{B} B$ |

which is obtained from the first order partial derivative of the interaction matrix of the non-linear system. The columns show how each variable affects each of the variables in the system. The matrix J thus represents the weighted topology of the interaction matrix of the system after linearization via the first terms of a Taylor series expansion (Hartman-Grobman theorem). The J matrix also allows evaluating the local stability of equilibrium points through the calculation of its eigenvalues. As also done in Moore et al. (2004), who follow classical ecological theory ([May, 1974](#_ENREF_4); [Pimm, 1984](#_ENREF_6); [Allesina and Tang, 2012](#_ENREF_1)) we estimated return time to equilibrium after perturbation as $-1/{{real(\lambda}_{max})}$, where ${real(\lambda}_{max})$ is the real part of the largest eigenvalue of J. The largest eigenvalue is used because in the linearized system the temporal (*t*) trajectory of the perturbation of each variable *x* (e.g. R or F) is of the form *x(t)* = *exp(ρ t) A* cos(*ω t* + *k*), where *ρ* and *ω* respectively represent the real and imaginary part of the eigenvalue *λ..* If *ρ* is negative for all the eigenvalues of J, all perturbations eventually decay.

We evaluated the Jacobian at the long-term equilibrium. Equilibrium was estimated by the state of the system after 10000 time steps, which was achieved by numerical integration of the system. We started by exploring the system with random sets of parameters sampled from a normal distribution with mean = 0.001 and sd = 0.1. However, a high fraction of the feasible (R, L, F and B all positive) and just randomly parameterised systems displayed long-term stability, as also confirmed by the stability analysis of the linearized systems (not shown here, for an example see R and MatLab scripts in the Supplementary Files). We then selected some combinations of parameters that generated reasonable final equilibrium states: for example, systems in which the long term dynamics lead to relatively low labile to recalcitrant pool ratios or systems with fungal to bacterial ratios that are in the range of what observed in the field. An example of such a combination of parameters (values rounded and adjusted to improve readability) is:

| $\lambda_{R}$ | *a* | | | *b* | | *db* | | *df* | | | $\alpha$ | | | *c* | | | | |  |  |  |
| --- | --- | --- | --- | --- | --- | --- | --- | --- | --- | --- | --- | --- | --- | --- | --- | --- | --- | --- | --- | --- | --- |
| *0.8* | *0.04* | | | *0.08* | | *0.01* | | *0.001* | | | *0.0005* | | | *0.0001* | | |  | |  |  |  |
|  | | |  | | |  | | |  | |  | |  | | |  | | |  | |  |
| *rf* | | $\lambda_{L}$ | | | *d* | | *e* | | | *rb* | | *rfl* | | | *mf* | | | *mb* | | | |
| *0.001* | | *0.1* | | | *0.1* | | *0.01* | | | *0.001* | | *0.01* | | | *0.01* | | | *0.001* | | | |

We started generating the scenarios shown in Fig. 1 (panel c-f) from similar sets of parameters, in which we varied the ratio between bacterial and fungal labile pool consumption rates. In panels c-f of Fig. 1, each dot is the long term equilibrium value of one scenario. The scenarios differ in $\lambda_{L}$, $\alpha$, $r_{B}$, and $r_{FL}$ with a positive correlation established between these parameters: low rates scenario had low values for these four rates and vice versa. See also Supplementary Files with R and MatLab scripts, which show how to solve one scenario and calculate the return time numerically.

**References**

Allesina, S., Tang, S., 2012. Stability criteria for complex ecosystems. Nature 483, 205-208.

Hale, J.K., 1980. Ordinary Differential Equations. Krieger, Malabar, FL.

Hartman, P., 1973. Ordinary Differential Equations, 2^nd^ ed. SIAM. Birkhäuser, Boston.

May, R.M., 1974. GENERAL INTRODUCTION TO ECOLOGICAL STABILITY.

Moore, J.C., Berlow, E.L., Coleman, D.C., de Ruiter, P.C., Dong, Q., Hastings, A., Johnson, N.C., McCann, K.S., Melville, K., Morin, P.J., Nadelhoffer, K., Rosemond, A.D., Post, D.M., Sabo, J.L., Scow, K.M., Vanni, M.J., Wall, D.H., 2004. Detritus, trophic dynamics and biodiversity. Ecology Letters 7, 584-600.

Pimm, S.L., 1984. The complexity and stability of ecosystems. Nature 307, 321-326.
